# The effect of triangular and reversed triangular flap designs to post third molar odontectomy complications (a pilot study)

**DOI:** 10.4317/jced.55864

**Published:** 2020-04-01

**Authors:** Henri Mudjono, Poerwati-Soetji Rahajoe, Elizabeth-Riyati-Titi Astuti

**Affiliations:** 1 Resident, Oral and Maxillofacial Surgery, Faculty of Dentistry, Gadjah Mada University; 2 Consultant and Lecturer, Oral and Maxillofacial Surgery, Faculty of Dentistry, Gadjah Mada University; 3 Lecturer, Oral and Maxillofacial Surgery, Faculty of Dentistry, Gadjah Mada University

## Abstract

**Background:**

Odontectomy is one of the most common surgical procedures in oral and maxillofacial surgery. Flap design influences the post operative complications. Triangular flap is the most widely used flap design but it has many shortages such as dehiscence, alveolar osteitis, reactionary bleeding, and periodontal disruption distal of second molar. The aim of this study is to introduce an alternative flap design in the surgical removal of impacted mandibular third molars – reversed triangular flap – and to compare this flap design with the triangular flap in case of dehiscence, reactionary bleeding, and clinical attachment loss.

**Material and Methods:**

This prospective, split-mouth study involved 15 patients with bilateral partially impacted mandibular third molars with similar impaction classification. One impacted tooth was removed using a triangular flap and the other using a reversed triangular flap. Post operative complications such as dehiscence, reactionary bleeding, and clinical attachment loss were recorded 1, 3, 7, 14, and 30 days post odontectomy.

**Results:**

Chi square test result shows that there were fewer incidences of dehiscence seven days post surgery using the reversed triangular flap (*p*=0.032). Mann Whitney-U test result shows that the reversed triangular flap exhibited less bleeding score on day 1 (*p*=0.002) and day 2 (*p*=0.035) post surgery. There were no statistically significant differences according to Mann Whitney-U test between the flap designs for the clinical attachment loss on distal of second molar on day 14 (*p*=0.512) and day 30 (*p*=0.902) post surgery.

**Conclusions:**

The reversed triangular flap design is preferable to triangular flap for impacted third molar surgery, escpecially in terms of wound dehisence and reactionary bleeding.

** Key words:**Flap design, third molar impaction, odontectomy, post odontectomy complications.

## Introduction

Odontectomy is one of the most frequently performed procedures in many oral and maxillofacial surgery practices (1). Morbidites that occur after odontectomy such as pain, swelling, trismus, alveolar osteitis, dehiscence, bleeding and compromised periodontal status of the preceding second molar are still haunting both the surgeon and patient. Flap design to expose the impacted tooth is one of the important determining factor (2-6). Triangular flap is the most common approach used by many surgeons. The disadvantage is that the triangular flap has difficulty to achieve primary wound closure on healthy bones, making it possible for dehiscence and complications such as alveolar osteitis. Some researchers still find the incidence of dehiscence in as much as 29.2 - 68% of the cases and alveolar osteitis as much as 10.8% using triangular flap design (3,7). Occurrence of oozing after odontectomy using triangular flap design is quite common (8). Periodontal health of the preceding second molar using triangular flap design has been studied with varying results (9,10).

As there are still disadvantages of the triangular flap design, the authors propose an alternative flap design, namely the reversed triangular flap design which is a modified pedicle flap design. The reversed triangular flap design starts with a vertical incision from the distal of the third molar towards the vestibule and rotates the flap to the disto-lingual side of the second molar. It is proposed that wound closure would occur primarily, making it easier to maintain wound hygiene, facilitating blood clotting, and would prevent dehiscence, thereby reducing the possibility of alveolar osteitis, soft tissue abscesses and would give benefit in patients using bisphosphonate, anti-coagulant, immunocompromised, and those undergoing radiotherapy (2,7).

This study aims to introduce an alternative flap design for odontectomy, namely a reversed triangular flap and compare it with the commonly used flap, namely the triangular flap. The reversed triangular flap is expected to reduce post odontectomy dehiscence, blood oozing, and clinical attachment loss.

## Material and Methods

-Patient selection

This study is a prospective and split-mouth clinical study. The research was conducted at the UGM Dental Hospital, Jogjakarta, Indonesia, involving 15 patients who had bilateral third molar impaction with similar classification. Each subject underwent two odontectomies with different flap technique, triang and rev tri. Before odontectomy, each subject was informed about the research protocol and signed an agreement to take part in the study. This study obtained a research approval from the UGM dental faculty´s ethics commission (001388 / KKEP / FKG-UGM / EC / 2018). The inclusion criterias were 18-30 years old, 10-60 minutes duration of operation, no history of systemic disease and drug allergy, not pregnant, not breastfeeding, or using contraceptive drugs, and no history of periodontal disease. Exclusion criterias were massive bleeding during the operation, drop out of the study, and the duration of the operation exceeded 60 minutes. Odontectomy was carried out by one operator (HM) and the observation of research parameters was carried out by one observer (LM). The patient chose the right or left side first to do odontectomy, the operator drew the type of flap used. A second odontectomy was performed at least one week after the first odontectomy.

-Surgical Procedure

Before odontectomy, the patient rinsed with a solution of diluted povidone iodine for 30 seconds. Lidocaine 2% with epinephrine 1: 80,000 (Pehacaine, PT. Kimia Farma, Jakarta, Indonesia) was used as a local anesthetic agent for mandibular and lingual nerve block and infiltration for buccal nerve.

**Figure 1:**
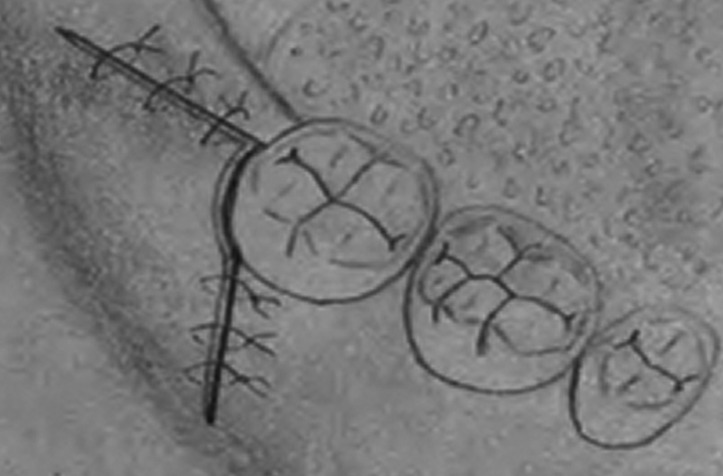
Suturing of triangular flap.

The triangular flap was made using an incision blade no.15 (Aesculap AG, Tuttlingen, Germany) from the mandibular ramus to disto-buccal side of the second molar, followed by an incision perpendicular to the mandibular vestibule approximately 10 mm (Fig. 1). Full thickness flap was raised and at the end of the operation, the flap was sutured to the lingual gingiva, adjacent to the distolingual line angle of the second molar. Suturing was continued on the distal side of the first suture and on the buccal side vertical incision.

**Figure 2:**
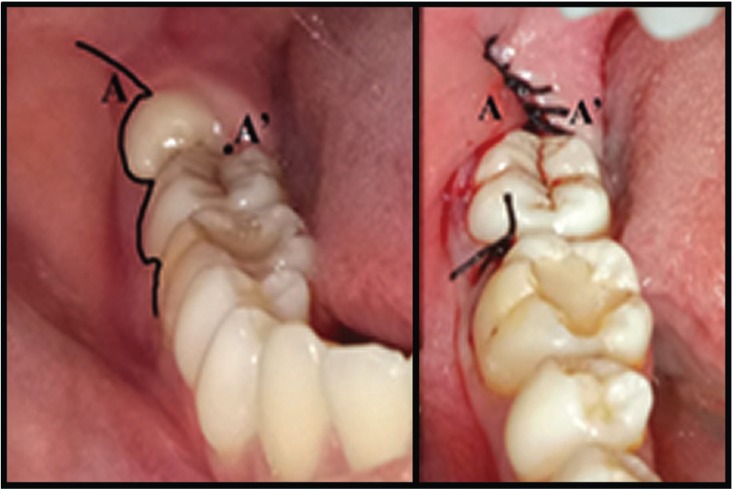
a) Flap design, and b) closure of reversed triangular flap.

The reversed triangular flap was also a full thickness flap and made with incision from the mesio-buccal angle of the first molar along the gingival sulcus to the disto-buccal angle of the second molar. The 90o vertical incision started from the distal mucosa which covers the third molar towards the vestibule approximately 10 mm (Fig. 2). Before flap closure was carried out, flap dissection had to be done so that the flap closure be obtained without tension. Flap closure was done by rotating the side of the buccal flap toward the disto-lingual side of the second molar, so that it could close the socket perfectly. Suturing started from the disto-lingual side of the lower second molar, proceeding distally until the distal end of the flap.

Osteotomy was done using surgery motor and straight handpiece with maximum speed 40.000 rpm (Surgic AP, Nakanishi Inc., Tochigi, Japan) and round bur (91002, SS White, New Jersey, USA) with 0.9% NaCl solution irigation. When necessary, the tooth was cut using Lindemann bur (SS White, New Jersey, USA) and was extracted using elevator and forceps (Kohler Medizintechnik, Stockach, Germany). Suturing was done using 4.0 silk suture (Triton, Medihop, Jakarta, Indonesia). The duration of the operation was calculated from the start of the incision to the completion of suturing. Post odontectomy oral medication was amoxicillin 500 mg 3x1 and ibuprofen 400 mg 2x1 for 5 days. 

-Postoperative Assessment

Dehiscence was observed on day 1, day 3, and day 7 post odontectomy. If dehiscence had happened, measurements were made using a caliper (Straumann, Basel, Switzerland). The length of dehiscence was measured from the distal of the second molar to the most distal opened incision, while the width of the dehiscence was measured from the widest incision line that opened from the buccal side to the lingual. Bleeding observations were carried out using visual analog score (VAS) according to Ghoreishian *et al.* (11). Bleeding VAS was assessed by the study subjects at the 1st hour, 6th hour, day 1 and day 2 post odontectomy. Clinical attachment loss (CAL) measurement was carried out on day 14 and 30 post odontectomy using the UNC-15 periodontal probe (Osung MND Co. Ltd., Gimpo-si, Gyeonggi-do, South Korea) at the bucco-distal line angle of the second molar. Measurements were calculated from the base of the gingival sulcus to the cemento-enamel junction in millimeters.

-Statistical Analysis

Data was analyzed using the IBM SPSS version 23.0 (IBM Corp., Armonk, NY, USA). Each parameter was analyzed using the Shapiro-Wilk and Levene test to determine normality and homogenity. The dehiscense frequency was analyzed using the chi-square test. The differences between overall observation were analyzed using the Friedmann test and the differences between individual observation time were analyzed using the Wilcoxson test. Differences between groups of observations were analyzed using the Mann Whitney-U test.

## Results

-Subject Characteristic

A total of 15 patients (9 males and 6 females) aged 19-26 became the subject of this study. Impaction classes were all class IA, and most of them were mesio-angular as much as 53.3%. All odontectomy flap was closed primarily.

-Dehisence

Dehiscence was not found in all subjects on day 1, and began to occur on day 3 in both groups with no significant differences. Dehiscence occurred 100% on day 7 in triangular flap group with significant differences compared to the reversed triangular flap group (Table 1).

**Table 1:**
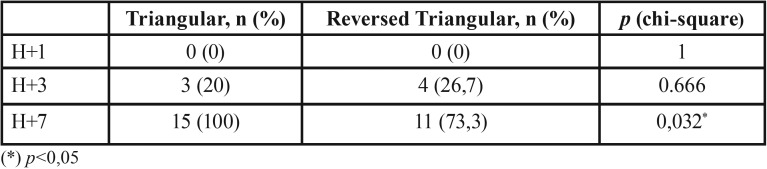
The number of dehiscence in each study group based on the observation day.

**Figure 3:**
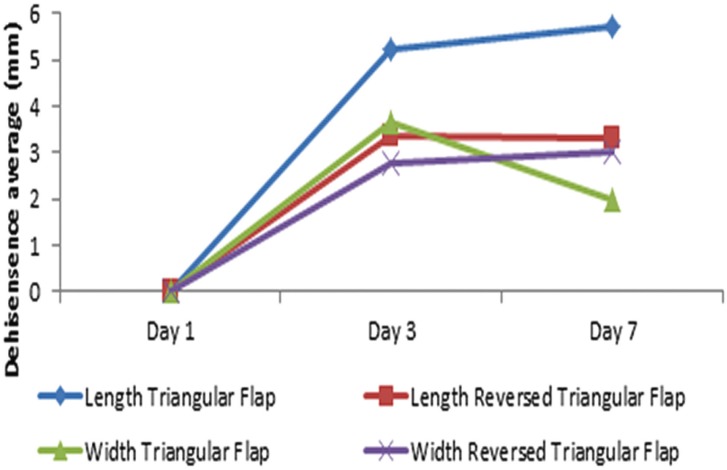
Average dehiscence length and width in triangular and reversed triangular flap design based on observation time.

The result of the measurement of the length of dehiscence shows the average dehiscence length in the triangular flap group is longer, both on day 3 and day 7 (Fig. 3) with significant difference on day 7 (Table 2). The longest value is found on day 7 triangular flap group. The dehiscence width on day 7 of the triangular flap group is lower than on day 3, with the highest dehiscence width is found on day 7 reversed triangular flap group (Fig. 3).

**Table 2:**
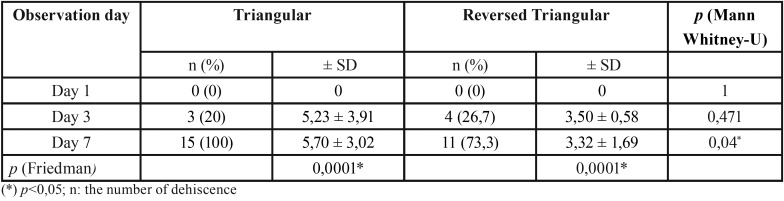
The dehiscence length in triangular flap and reversed triangular flap design.

The Friedman test results (Table 2) show that there are significant differences in wound dehiscence both the length and width of the two groups on day 1, day 3, and day 7 (*p* <0.05). The Wilcoxon test results showed significant differences (*p* <0.05) in the length and width of wound dehiscence between day 1 and day 7 in both groups. The results of the Mann Whitney-U test (Table 2) showed significant difference only occur in the length of wound dehiscence between the reversed triangular flap compared to the triangular flap at day 7 (*p* <0.05).

-Bleeding

The measurement results for post-odontectomy bleeding VAS (Table 3) showed that the maximal bleeding score in the reversed triangular flap group was lower than the triangular flap both on 1st hour, 6th hour, day 1, and day 2. The highest score for the triangular and reversed triangular flap group 1 hour post odontectomy was score 1, 46.7% and 40% respectively. Likewise, the observation 6 hours post odontectomy was 60% in the triangular group and 46.7% in the reversed triangular group. The results of the Mann Whitney-U test at 1 hour and 6 hours post odontectomy showed no significant differences between the two groups (Table 3).

**Table 3:**
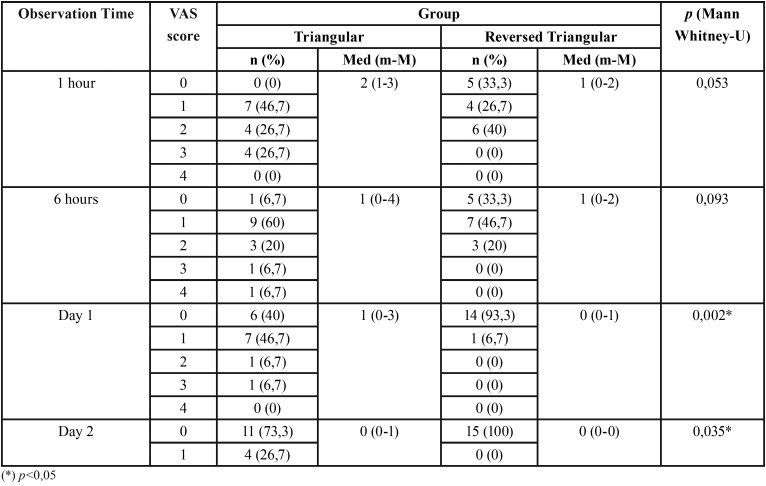
Frequency of post-odontectomy bleeding VAS score in triangular and reversed triangular flap group.

Observation of day 1 post odontectomy in the triangular reversed flap group showed a decrease in VAS scores, with the highest score being 0 (93.3%), while the highest score in the triangular flap design group was a score of 1 (46.7%) with significant difference (*p* = 0.002). All research subjects in the reversed triangular flap group at day 2 showed no bleeding (VAS = 0), while in the triangular flap group 73.3% with significant difference (*p* = 0.035).

-Clinical Attachment Loss

The CAL measurement results (Table 4) show that the highest CAL average value in the reversed triangular flap group was found at day 14, and the lowest in the triangular flap design group found at day 30.

**Table 4:**
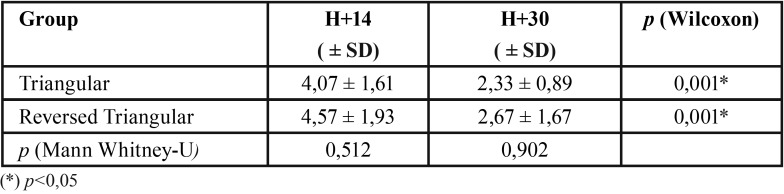
CAL value in triangular flap and reversed triangular flap groups.

The CAL value in the two flap design groups has the same pattern which is reduced according to the observation time. The Wilcoxon test results showed that there was a significant decrease in the average CAL from day 14 to day 30 in both groups (*p* <0.05). There were no significant differences in both groups for day 14 and day 30 (*p*> 0.05).

## Discussion

This study design was split mouth with similar impaction classification. The advantage of split mouth design is that every subject becomes control for him/herself in order to avoid bias and inter subject variability. Odontectomy is carried out by one operator and observation of research parameters is carried out by another observer (LM). Many clinical trials were done using split mouth design (3,5-7,10).

Triangular and envelope flaps were the most studied flap design with various variables such as pain, swelling, trismus, dehiscence, and periodontal health (2,9,10,12-18). Reversed triangular flap was an alternative flap and differs from triangular flap in the position of its vertical incision which was located on distal of third molar. Flap closure was done by rotating the side of the buccal flap toward the disto-lingual side of the second molar, so that it could close the socket completely.

Post odontectomy dehiscence mostly happens because of inadequate mucosa for tension free approximation of buccal and lingual mucosa for primary closure of the surgical wound (17). Dehiscence began to happen on day 3 in both groups with no difference between the triangular and reversed triangular flap groups at the incidence (*p* = 0.666), length (*p* = 0.471) and width of dehiscence (*p* = 0.271).

The dehiscence incidence on day 7 occurred as much as 100% in the triangular flap group, while the reversed triangular flap design group also had a 46.7% incidence (*p* = 0.032). Various results happened when observing dehiscence using triangular flap on day 7. Jakse *et al*. (2) found that wound dehiscence developed in only 10% of cases in which the modified triangular flap dibandingkan dgn envelope was used. Desai *et al.* (16) reported 60% dehiscence using triangular flap compared with envelope flap. The same result was also stated by Monaco *et al.* (14) Comparing triangular flap with lingually based flap, Yolcu and Acar (7) also reported 68% dehiscence using triangular flap. Rakprasitkul and Pairuchvej (19) found that 73% of the dehiscence occured in triangular flap with primary closure on day 7 post-odontectomy, but odontectomy was performed on all impaction classifications and was not compared with reversed triangular flap design. In contrast to this study is that all research subjects had IA impaction classification which has bigger gap between third and second molar.

All subject of the triangular flap group experienced dehiscence could be due to lack of mucosa used to close the extraction socket so that there was tension when suturing the buccal flap with the lingual part of partially impacted third molar (3,20). Several other studies (2,17) found that dehiscence in triangular flap design was less than envelope flap design, but we found no studies comparing triangular flap with reversed triangular flap design.

The reversed triangular flap gave less dehiscence results than the triangular flap with a significant difference in the average dehiscence length at day 7 (*p* = 0.04) between the two groups (Table 2). Other researchers found the same thing, there were still 27% dehiscence using pedicle flap, which is almost similar with reversed triangular, compared with envelope flap on day 7 because there was still tension on the flap (21). Reversed triangular flap could reduce tension and dehiscence from wider dissection of the flap to the distal and inferior. Another type of pedicle flap, the mesial papilla-sparing marginal incision, was also considered as a desirable surgical technique that gave less dehiscence compared with triangular and envelope flap (3).

Bleeding observed in this study was local bleeding (oozing) which caused from local factor such as flap design. Systemic factor had been excluded from this study. There had been few study that observed post odontectomy bleeding. This study used visual analog score according to Ghoreishian *et al.* (11) Other study on post odontectomy bleeding was also using the same scoring system but excluded score 4 (massive bleeding) (22). Limitation of the subjective assessment is the difference in judgement between individuals. Therefore it is necessary to make an objective measurement to assess post-odontectomy bleeding.

Flap closure in the reversed triangular flap design occurred primarily, which was seen in the observation of the 1st and 6th hour post odontectomy in the reversed triangular flap group, there were 33.3% of all study subjects who did not experience bleeding (VAS=0). Setiya *et al.* stated that all of the study subjects still experienced oozing on the 1st day with triangular flap (8). Oozing events at the 1st and 6th hours observations of the triangular reversed flap group were less than the triangular flap design group (Table 3), and there were no significant differences between the two groups (*p*> 0.05). Oozing is mostly observed where vertical incisions are located in the triangular flap design, which happens because there is a disruption in the edges of the wound due to jaw movement and mastication. Bleeding can be minimized by using a good surgical technique and by avoiding the tearing of flaps or excessive trauma to bone and the overlying soft tissue (23)

The difference between the two flap designs began to be observed at day 1 which showed that 60% of the research subjects still felt blood oozing (VAS = 1) in the triangular flap design group. This result consistents with the report from Joshi *et al*., in 33.3% of the study subjects (22). In contrast, in the reversed triangular flap group, only one study subject (6.67%) assessed blood oozing in day 1, with a significant difference between the two groups (*p* = 0.002). The hemostasis process occurs faster in reversed triangular flap design than triangular flap design because primary closure facilitates the formation of coagulum.

Periodontal disruption is a common complication post odontectomy. Various research comparing the effect of different flaps to periodontal health using various parameters with various results (6,9,10,15). This study showed that the value of CAL decreased with the time of observation, with the value in the triangular reversed flap design group higher than the triangular flap design group, both at day 14 and day 30, and there were significant differences (*p* = 0.001) between the two groups (Table 4). The reduced value of CAL observed up to 6 months after odontectomy using the triangular flap design was also encountered by Chang *et al.* (24) and Leung *et al.* (25). The limitation of this study was that no CAL measurements had been taken before odontectomy so that the condition of periodontal tissue before odontectomy was unknown. The CAL value in the reversed triangular flap group was higher because the gingival sulcus incision was longer and the more bone surface was reduced during osteotomy than the triangular flap design, causing disruption to the periodontal tissue and increasing bone resorption due to increased osteoclast activity (26). Therefore the triangular flap is preferable for deeper impaction.

The average value of CAL day 30 in both groups was still above the normal value of the gingival sulcus (> 2 mm) which means that the process of healing periodontal tissue is still ongoing. The remodeling phase starts 21 days after the trauma and the strength of the collagen matrix reaches 20% within 30 days after trauma (27).

The average value of CAL did not show a significant difference between the two types of flap design both on the observations on day 14 (*p* = 0.865) and day 30 (*p* = 0.676). Kiritoglu *et al.*(10) also stated that there was no difference in probing depth between modified triangular and 3-cornered flap on four weeks observation. Other study, comparing the design of triangular and bayonet flaps, suggests that there is no difference in CAL values of 6 months and 12 months post-odontectomy (9). Another meta-analysis study shows that surgical extraction of impacted third molar with different flap designs seems to have no significant impact on the periodontal condition of the adjacent second molar (28). However longer observation day until day 90 shows that there is correlation between probing depth and flap design using triangular and envelope flap (6,29). At present there is no other study comparing the value of CAL between triangular and reversed triangular flap designs.

The results of the study on day 14 and day 30 did not show any significant difference because the healing of periodontal tissue was not complete. The wound maturation phase in the periodontal tissue will be completed one year after the trauma (30). The addition of observation time to the end of the healing of the periodontal tissue is needed to get better results.

Limitation of this study is the small number of subjects involved in due to the difficulty in obtaining subjects who have bilateral third molar impaction with similar classification. Nevertheless this study has met the requirements of a minimum number of clinical research samples. Increasing the number of research subject would improve the quality of the study.

## Conclusions

Based on the results of research and data analysis it can be concluded that the incidence of dehiscence in the reversed triangular flap group was less than the triangular flap design group, especially day 7 after odontectomy. Post-odontectomy bleeding stopped faster in the reversed triangular flap group compared to the triangular flap group. Flap design does not affect the clinical attachment loss value distal of the second molar on day 14 and day 30 after third molar odontectomy.
